# (2*E*)-1-(Pyridin-2-yl)-3-(2,4,6-trimeth­oxy­phen­yl)prop-2-en-1-one

**DOI:** 10.1107/S1600536811039110

**Published:** 2011-09-30

**Authors:** Hoong-Kun Fun, Suchada Chantrapromma, Thitipone Suwunwong

**Affiliations:** a X-ray Crystallography Unit, School of Physics, Universiti Sains Malaysia, 11800 USM, Penang, Malaysia; b Crystal Materials Research Unit, Department of Chemistry, Faculty of Science, Prince of Songkla University, Hat-Yai, Songkhla 90112, Thailand

## Abstract

The title heteroaryl chalcone derivative, C_17_H_17_NO_4_, is a condensation product of 2-acetyl­pyridine and 2,4,6-trimeth­oxy­benzaldehyde. The mol­ecule is roughly planar, the dihedral angle between the pyridine and benzene rings being 5.51 (10)°. All the three meth­oxy groups are almost co-planar with the bound benzene ring [r.m.s. deviation of 0.0306 (2) Å]. A weak C—H⋯O intra­molecular inter­action involving one of the *ortho*-meth­oxy groups generates an *S*(6) ring motif. In the crystal, the mol­ecules are linked by weak C—H⋯O inter­actions into anti-parallel face-to-face pairs. Adjacent pairs are further connected into sheets parallel to the *ab* plane.

## Related literature

For bond-length data, see: Allen *et al.* (1987 ▶). For hydrogen-bond motifs, see: Bernstein *et al.* (1995 ▶). For related structures, see: Chantrapromma *et al.* (2009 ▶); Fun *et al.* (2010 ▶, 2011 ▶). For background to and applications of chalcones and heteroaryl chalcones, see: Bandgar *et al.* (2010 ▶); Gacche *et al.* (2008 ▶); Go *et al.* (2005 ▶); Isomoto *et al.* (2005 ▶); Jung *et al.* (2008 ▶); Suwunwong *et al.* (2011 ▶); Tewtrakul *et al.* (2003 ▶). For the stability of the temperature controller used in the data collection, see Cosier & Glazer, (1986 ▶).
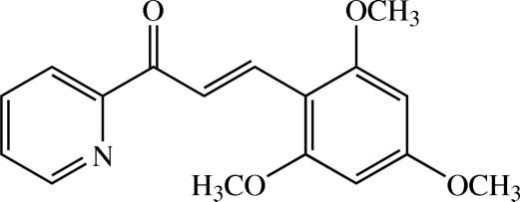


## Experimental

### 

#### Crystal data

C_17_H_17_NO_4_
*M* *_r_* = 299.32
Orthorhombic, 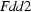

*a* = 31.563 (2) Å
*b* = 44.508 (3) Å
*c* = 3.9504 (3) Å
*V* = 5549.6 (7) Å^3^
*Z* = 16
Mo *K*α radiation
μ = 0.10 mm^−1^
*T* = 100 K
0.58 × 0.14 × 0.04 mm

#### Data collection

Bruker APEXII CCD area detector diffractometer
Absorption correction: multi-scan (*SADABS*; Bruker, 2005 ▶) *T*_min_ = 0.943, *T*_max_ = 0.996
31465 measured reflections
2309 independent reflections
1908 reflections with *I* > 2σ(*I*)
*R*_int_ = 0.100

#### Refinement

*R*[*F* ^2^ > 2σ(*F* ^2^)] = 0.043
*wR*(*F* ^2^) = 0.105
*S* = 1.09
2309 reflections
267 parameters
1 restraint
All H-atom parameters refined
Δρ_max_ = 0.23 e Å^−3^
Δρ_min_ = −0.27 e Å^−3^

### 

Data collection: *APEX2* (Bruker, 2005 ▶); cell refinement: *SAINT* (Bruker, 2005 ▶); data reduction: *SAINT*; program(s) used to solve structure: *SHELXTL* (Sheldrick, 2008 ▶); program(s) used to refine structure: *SHELXTL*; molecular graphics: *SHELXTL*; software used to prepare material for publication: *SHELXTL* and *PLATON* (Spek, 2009 ▶).

## Supplementary Material

Crystal structure: contains datablock(s) global, I. DOI: 10.1107/S1600536811039110/rz2642sup1.cif

Structure factors: contains datablock(s) I. DOI: 10.1107/S1600536811039110/rz2642Isup2.hkl

Supplementary material file. DOI: 10.1107/S1600536811039110/rz2642Isup3.cml

Additional supplementary materials:  crystallographic information; 3D view; checkCIF report

## Figures and Tables

**Table 1:** Hydrogen-bond geometry (Å, °)

*D*—H⋯*A*	*D*—H	H⋯*A*	*D*⋯*A*	*D*—H⋯*A*
C3—H3*A*⋯O2^i^	1.00 (2)	2.45 (3)	3.369 (2)	157.8 (17)
C7—H11*B*⋯O4	0.97 (3)	2.31 (2)	2.835 (2)	113.1 (18)
C17—H17*B*⋯O4^ii^	0.99 (2)	2.46 (2)	3.337 (3)	148 (2)

Symmetry codes: (i) 
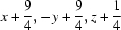
; (ii) 
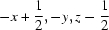
.

**Fig. 1.**
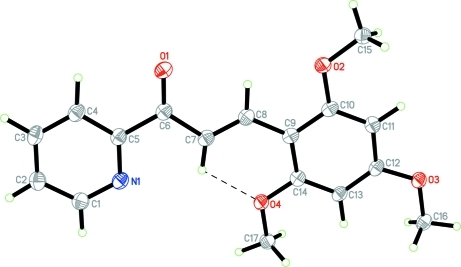
The molecular structure of the title compound, showing 50% probability displacement ellipsoids. A weak intramolecular C—H···O interaction is shown as a dashed line.

**Fig. 2.**
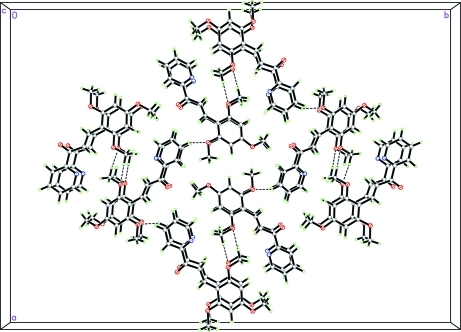
The crystal packing of the title compound viewed along the c axis, showing molecular sheets parallel to the ab plane. Hydrogen bonds are shown as dashed lines.

## supplementary crystallographic information

### Comment

Chalcones and heteroaryl chalcones have drawn a lot of interests due to their wide range of biological properties including antioxidant (Gacche *et al.*, 2008), antibacterial (Go *et al.*, 2005; Isomoto *et al.*, 2005), anti-inflammatory and anticancer (Bandgar *et al.*, 2010) as well as HIV-1 protease inhibitory (Tewtrakul *et al.*, 2003) activities. Furthermore they also exhibit fluorescent property (Jung *et al.*, 2008; Suwunwong *et al.*, 2011). In our on-going research on the biological and fluorescent properties of chalcones and heteroaryl chalcones (Chantrapromma *et al.*, 2009; Fun *et al.*, 2010, 2011; Suwunwong *et al.*, 2011), the title heteroaryl chalcone derivative (I) was synthesized in order to study the effects of substituted postions on the fluorescent property in comparision with the closely related compounds (Fun *et al.*, 2011; Suwunwong *et al.*, 2011). In addition (I) was also tested for analgesic and antibacterial activities. Our results showed that (I) exhibits a moderate analgesic activity but is inactive for antibacterial activity. Herein we report the crystal structure of (I).

The molecule of the title heteroaryl chalcone derivative (Fig. 1) exists in an *E* configuration with respect to the C7═C8 double bond [1.341 (3) Å]. The torsion angle C6–C7–C8–C9 is 179.0 (2)°. The molecule is almost planar with a dihedral angle between the pyridine and 2,4,6-trimethoxyphenyl rings of 5.51 (10)°. Atoms of the propenone bridge (C6, C7, C8 and O1) lie on the same plane [*r.m.s*. deviation of 0.017 (2)] and the torsion angle O1–C6–C7–C8 is -5.8 (4)°. The mean plane through this bridge makes dihedral angles of 6.96 (16) and 11.72 (16)° with the planes of pyridine and benzene rings, respectively. All the three substituted methoxy groups of the 2,4,6-trimethoxyphenyl unit are co-planar with the phenyl ring as indicated by the torsion angles C15–O2–C10–C11 = -0.4 (3)°, C16–O3–C12–C13 = 0.9 (3)° and C17–O4–C14–C13 = -4.7 (3)°. In the molecule, a weak intramolecular C7—H7A···O4 interaction (Table 1) generates an S(6) ring motif (Bernstein *et al.*, 1995). The bond distances are of normal values (Allen *et al.*, 1987) and comparable with related structures (Chantrapromma *et al.*, 2009; Fun *et al.*, 2010; 2011).

In the crystal packing (Fig. 2), only the two *ortho*-methoxy groups are involved in weak C—H···O interactions (Table 1). The adjacent molecules are linked by weak C17—H17B···O4 interaction (Table 1) into anti-parallel face-to-face pairs. The adjacent pairs were further connected by weak C3—H3A···O2 interactions (Table 1) into sheets parallel to the *ab* plane which are stacked down the *c* axis. The crystal may be further stabilized by C···O [3.203 (2) Å] short contacts.

### Experimental

The title compound was synthesized by the condensation reaction of 2,4,6-trimethoxybenzaldehyde (0.40 g, 2 mmol) with 2-acetylpyridine (0.20 g, 2 mmol) in ethanol (30 ml) in the presence of 30% NaOH(aq) (5 ml). After stirring in ice bath at 278 K for 4 h, the resulting pale yellow solid appeared and was then collected by filtration, washed with distilled water, dried and purified by repeated recrystallization from acetone. Pale yellow plate-shaped single crystals of the title compound suitable for *X*-ray structure determination were recrystalized from acetone/ethanol (1:1 *v*/*v*) by the evaporation of the solvent at room temperature after several days, M.p. 392-393 K.

### Refinement

All H atoms were located in difference maps and refined isotropically. A total of 1754 Friedel pairs were merged before final refinement as there is no large anomalous dispersion for the determination of the absolute structure.

### Crystal data

**Table:**

C_17_H_17_NO_4_	*D*_x_ = 1.433 Mg m^−^^3^
*M**_r_* = 299.32	Melting point = 392–393 K
Orthorhombic, *F**d**d*2	Mo *K*α radiation, λ = 0.71073 Å
Hall symbol: F 2 -2d	Cell parameters from 2309 reflections
*a* = 31.563 (2) Å	θ = 1.6–30.0°
*b* = 44.508 (3) Å	µ = 0.10 mm^−^^1^
*c* = 3.9504 (3) Å	*T* = 100 K
*V* = 5549.6 (7) Å^3^	Plate, pale yellow
*Z* = 16	0.58 × 0.14 × 0.04 mm
*F*(000) = 2528	

### Data collection

**Table:**

Bruker APEXII CCD area detector diffractometer	2309 independent reflections
Radiation source: sealed tube	1908 reflections with *I* > 2σ(*I*)
graphite	*R*_int_ = 0.100
φ and ω scans	θ_max_ = 30.0°, θ_min_ = 1.6°
Absorption correction: multi-scan (*SADABS*; Bruker, 2005)	*h* = −44→44
*T*_min_ = 0.943, *T*_max_ = 0.996	*k* = −62→62
31465 measured reflections	*l* = −5→5

### Refinement

**Table:**

Refinement on *F*^2^	Primary atom site location: structure-invariant direct methods
Least-squares matrix: full	Secondary atom site location: difference Fourier map
*R*[*F*^2^ > 2σ(*F*^2^)] = 0.043	Hydrogen site location: inferred from neighbouring sites
*wR*(*F*^2^) = 0.105	All H-atom parameters refined
*S* = 1.09	*w* = 1/[σ^2^(*F*_o_^2^) + (0.0467*P*)^2^ + 4.8268*P*] where *P* = (*F*_o_^2^ + 2*F*_c_^2^)/3
2309 reflections	(Δ/σ)_max_ = 0.001
267 parameters	Δρ_max_ = 0.23 e Å^−^^3^
1 restraint	Δρ_min_ = −0.27 e Å^−^^3^

### Special details

**Table:**

Experimental. The crystal was placed in the cold stream of an Oxford Cryosystems Cobra open-flow nitrogen cryostat (Cosier & Glazer, 1986) operating at 120.0 (1) K.
Geometry. All esds (except the esd in the dihedral angle between two l.s. planes) are estimated using the full covariance matrix. The cell esds are taken into account individually in the estimation of esds in distances, angles and torsion angles; correlations between esds in cell parameters are only used when they are defined by crystal symmetry. An approximate (isotropic) treatment of cell esds is used for estimating esds involving l.s. planes.
Refinement. Refinement of F^2^ against ALL reflections. The weighted R-factor wR and goodness of fit S are based on F^2^, conventional R-factors R are based on F, with F set to zero for negative F^2^. The threshold expression of F^2^ > 2sigma(F^2^) is used only for calculating R-factors(gt) etc. and is not relevant to the choice of reflections for refinement. R-factors based on F^2^ are statistically about twice as large as those based on F, and R- factors based on ALL data will be even larger.

### Fractional atomic coordinates and isotropic or equivalent isotropic displacement parameters (Å^2^)

**Table:**

	*x*	*y*	*z*	*U*_iso_*/*U*_eq_	
O1	0.18610 (5)	0.11217 (3)	1.2030 (5)	0.0298 (4)	
O2	0.07115 (4)	0.04799 (3)	1.1758 (5)	0.0239 (3)	
O3	0.05980 (4)	−0.04831 (3)	0.6752 (5)	0.0248 (4)	
O4	0.19307 (4)	0.00496 (3)	0.6918 (5)	0.0242 (4)	
N1	0.27419 (5)	0.08486 (4)	0.7393 (6)	0.0245 (4)	
C1	0.31419 (7)	0.09271 (5)	0.6662 (7)	0.0263 (5)	
C2	0.33299 (7)	0.11932 (5)	0.7701 (7)	0.0266 (5)	
C3	0.30912 (7)	0.13912 (5)	0.9612 (7)	0.0270 (5)	
C4	0.26779 (7)	0.13153 (5)	1.0406 (7)	0.0254 (5)	
C5	0.25139 (6)	0.10419 (4)	0.9256 (7)	0.0225 (4)	
C6	0.20685 (6)	0.09511 (4)	1.0251 (7)	0.0226 (4)	
C7	0.19132 (7)	0.06578 (4)	0.9048 (7)	0.0228 (4)	
C8	0.15391 (7)	0.05521 (4)	1.0129 (7)	0.0228 (4)	
C9	0.13222 (6)	0.02737 (4)	0.9251 (6)	0.0213 (4)	
C10	0.08883 (6)	0.02411 (4)	1.0100 (6)	0.0217 (4)	
C11	0.06569 (6)	−0.00125 (4)	0.9267 (7)	0.0225 (4)	
C12	0.08555 (6)	−0.02465 (4)	0.7554 (6)	0.0216 (4)	
C13	0.12828 (6)	−0.02332 (4)	0.6760 (7)	0.0220 (4)	
C14	0.15094 (6)	0.00253 (4)	0.7601 (6)	0.0217 (4)	
C15	0.02726 (7)	0.04603 (5)	1.2600 (7)	0.0252 (5)	
C16	0.07863 (7)	−0.07331 (5)	0.5027 (7)	0.0259 (5)	
C17	0.21410 (7)	−0.02095 (5)	0.5537 (7)	0.0261 (5)	
H1A	0.3305 (7)	0.0785 (5)	0.524 (8)	0.023 (6)*	
H2A	0.3622 (7)	0.1239 (5)	0.708 (9)	0.027 (6)*	
H3A	0.3216 (7)	0.1581 (5)	1.051 (8)	0.025 (6)*	
H4A	0.2489 (7)	0.1445 (5)	1.187 (9)	0.030 (7)*	
H8A	0.1375 (8)	0.0676 (6)	1.166 (9)	0.039 (8)*	
H11A	0.0350 (7)	−0.0029 (5)	0.980 (8)	0.023 (6)*	
H11B	0.2095 (7)	0.0554 (5)	0.747 (9)	0.025 (6)*	
H13A	0.1415 (7)	−0.0402 (5)	0.545 (8)	0.025 (6)*	
H15A	0.0101 (8)	0.0457 (6)	1.054 (9)	0.033 (8)*	
H15B	0.0223 (8)	0.0281 (6)	1.428 (10)	0.038 (7)*	
H15C	0.0194 (7)	0.0634 (5)	1.389 (8)	0.024 (7)*	
H16A	0.0549 (7)	−0.0874 (5)	0.453 (8)	0.023 (6)*	
H16B	0.0916 (7)	−0.0668 (5)	0.282 (9)	0.023 (6)*	
H16C	0.1003 (8)	−0.0827 (5)	0.644 (9)	0.031 (7)*	
H17A	0.2010 (8)	−0.0257 (6)	0.328 (9)	0.030 (8)*	
H17B	0.2445 (7)	−0.0160 (5)	0.542 (8)	0.025 (6)*	
H17C	0.2105 (7)	−0.0383 (5)	0.714 (9)	0.028 (7)*	

### Atomic displacement parameters (Å^2^)

**Table:**

	*U*^11^	*U*^22^	*U*^33^	*U*^12^	*U*^13^	*U*^23^
O1	0.0320 (8)	0.0216 (7)	0.0360 (11)	−0.0005 (6)	0.0043 (8)	−0.0058 (8)
O2	0.0246 (7)	0.0189 (6)	0.0283 (9)	0.0001 (5)	0.0026 (7)	−0.0024 (7)
O3	0.0251 (7)	0.0175 (6)	0.0317 (10)	−0.0026 (5)	0.0018 (7)	−0.0032 (7)
O4	0.0236 (7)	0.0194 (6)	0.0295 (10)	−0.0009 (5)	0.0026 (7)	−0.0034 (7)
N1	0.0284 (9)	0.0189 (8)	0.0264 (11)	0.0003 (6)	−0.0011 (8)	−0.0007 (8)
C1	0.0279 (10)	0.0233 (9)	0.0276 (13)	0.0008 (8)	0.0002 (10)	0.0019 (10)
C2	0.0290 (11)	0.0239 (9)	0.0270 (13)	−0.0012 (8)	−0.0014 (10)	0.0061 (10)
C3	0.0320 (11)	0.0203 (9)	0.0288 (14)	−0.0037 (8)	−0.0050 (11)	0.0023 (10)
C4	0.0313 (11)	0.0197 (9)	0.0251 (12)	−0.0004 (8)	−0.0021 (10)	0.0009 (9)
C5	0.0274 (10)	0.0188 (9)	0.0214 (11)	−0.0013 (7)	−0.0012 (9)	0.0033 (9)
C6	0.0267 (10)	0.0185 (9)	0.0224 (11)	0.0004 (7)	−0.0010 (9)	0.0029 (9)
C7	0.0278 (10)	0.0162 (9)	0.0242 (12)	0.0008 (7)	−0.0007 (9)	0.0012 (9)
C8	0.0266 (10)	0.0173 (8)	0.0245 (12)	0.0013 (7)	−0.0014 (9)	0.0017 (9)
C9	0.0258 (10)	0.0181 (9)	0.0200 (12)	0.0013 (7)	−0.0009 (9)	0.0019 (9)
C10	0.0287 (10)	0.0162 (8)	0.0201 (11)	0.0015 (7)	−0.0017 (9)	0.0011 (9)
C11	0.0233 (9)	0.0195 (9)	0.0248 (12)	0.0001 (7)	0.0002 (9)	0.0027 (9)
C12	0.0287 (10)	0.0156 (8)	0.0206 (12)	−0.0024 (7)	−0.0039 (9)	0.0021 (8)
C13	0.0263 (10)	0.0176 (8)	0.0220 (12)	−0.0009 (7)	−0.0012 (9)	−0.0009 (9)
C14	0.0245 (10)	0.0191 (9)	0.0214 (12)	0.0007 (7)	−0.0014 (9)	0.0028 (9)
C15	0.0247 (10)	0.0231 (9)	0.0279 (13)	0.0018 (8)	0.0003 (10)	−0.0022 (10)
C16	0.0305 (11)	0.0179 (9)	0.0292 (13)	−0.0016 (8)	−0.0011 (10)	−0.0026 (10)
C17	0.0264 (11)	0.0194 (9)	0.0325 (14)	0.0009 (8)	0.0045 (10)	−0.0029 (10)

### Geometric parameters (Å, °)

**Table:**

O1—C6	1.224 (3)	C7—H11B	0.96 (3)
O2—C10	1.368 (2)	C8—C9	1.458 (3)
O2—C15	1.427 (3)	C8—H8A	0.97 (3)
O3—C12	1.367 (2)	C9—C14	1.413 (3)
O3—C16	1.434 (3)	C9—C10	1.417 (3)
O4—C14	1.361 (2)	C10—C11	1.384 (3)
O4—C17	1.438 (3)	C11—C12	1.392 (3)
N1—C5	1.341 (3)	C11—H11A	0.99 (2)
N1—C1	1.342 (3)	C12—C13	1.386 (3)
C1—C2	1.387 (3)	C13—C14	1.395 (3)
C1—H1A	0.99 (3)	C13—H13A	1.00 (3)
C2—C3	1.383 (3)	C15—H15A	0.98 (3)
C2—H2A	0.97 (2)	C15—H15B	1.05 (3)
C3—C4	1.384 (3)	C15—H15C	0.96 (3)
C3—H3A	1.00 (2)	C16—H16A	1.00 (2)
C4—C5	1.398 (3)	C16—H16B	1.01 (3)
C4—H4A	1.01 (3)	C16—H16C	0.98 (3)
C5—C6	1.515 (3)	C17—H17A	1.01 (3)
C6—C7	1.474 (3)	C17—H17B	0.99 (2)
C7—C8	1.341 (3)	C17—H17C	1.00 (3)
			
C10—O2—C15	117.39 (16)	O2—C10—C9	115.35 (17)
C12—O3—C16	117.48 (16)	C11—C10—C9	122.49 (19)
C14—O4—C17	117.55 (16)	C10—C11—C12	119.22 (19)
C5—N1—C1	117.13 (18)	C10—C11—H11A	121.7 (14)
N1—C1—C2	124.1 (2)	C12—C11—H11A	119.1 (14)
N1—C1—H1A	116.3 (13)	O3—C12—C13	123.98 (18)
C2—C1—H1A	119.5 (13)	O3—C12—C11	114.91 (18)
C3—C2—C1	118.2 (2)	C13—C12—C11	121.11 (18)
C3—C2—H2A	121.2 (15)	C12—C13—C14	118.69 (19)
C1—C2—H2A	120.6 (15)	C12—C13—H13A	119.3 (13)
C2—C3—C4	118.8 (2)	C14—C13—H13A	121.8 (13)
C2—C3—H3A	121.3 (14)	O4—C14—C13	121.27 (19)
C4—C3—H3A	119.8 (14)	O4—C14—C9	115.97 (17)
C3—C4—C5	119.2 (2)	C13—C14—C9	122.75 (18)
C3—C4—H4A	123.1 (13)	O2—C15—H15A	110.2 (17)
C5—C4—H4A	117.7 (13)	O2—C15—H15B	109.8 (14)
N1—C5—C4	122.54 (19)	H15A—C15—H15B	115 (2)
N1—C5—C6	117.99 (17)	O2—C15—H15C	109.0 (14)
C4—C5—C6	119.4 (2)	H15A—C15—H15C	108 (2)
O1—C6—C7	123.82 (19)	H15B—C15—H15C	104 (2)
O1—C6—C5	118.70 (18)	O3—C16—H16A	105.8 (14)
C7—C6—C5	117.47 (19)	O3—C16—H16B	111.0 (14)
C8—C7—C6	120.1 (2)	H16A—C16—H16B	108 (2)
C8—C7—H11B	124.1 (14)	O3—C16—H16C	110.5 (17)
C6—C7—H11B	115.8 (14)	H16A—C16—H16C	112 (2)
C7—C8—C9	129.5 (2)	H16B—C16—H16C	109 (2)
C7—C8—H8A	118.2 (15)	O4—C17—H17A	108.4 (15)
C9—C8—H8A	112.3 (15)	O4—C17—H17B	106.7 (14)
C14—C9—C10	115.68 (17)	H17A—C17—H17B	114 (2)
C14—C9—C8	125.35 (18)	O4—C17—H17C	109.0 (16)
C10—C9—C8	118.97 (18)	H17A—C17—H17C	111 (2)
O2—C10—C11	122.16 (18)	H17B—C17—H17C	108 (2)
			
C5—N1—C1—C2	−0.2 (4)	C8—C9—C10—O2	−1.0 (3)
N1—C1—C2—C3	0.0 (4)	C14—C9—C10—C11	−2.2 (3)
C1—C2—C3—C4	0.1 (4)	C8—C9—C10—C11	178.1 (2)
C2—C3—C4—C5	−0.1 (4)	O2—C10—C11—C12	179.5 (2)
C1—N1—C5—C4	0.3 (3)	C9—C10—C11—C12	0.5 (4)
C1—N1—C5—C6	−177.1 (2)	C16—O3—C12—C13	0.9 (3)
C3—C4—C5—N1	−0.1 (4)	C16—O3—C12—C11	−179.1 (2)
C3—C4—C5—C6	177.2 (2)	C10—C11—C12—O3	−178.1 (2)
N1—C5—C6—O1	178.0 (2)	C10—C11—C12—C13	1.9 (4)
C4—C5—C6—O1	0.5 (3)	O3—C12—C13—C14	177.6 (2)
N1—C5—C6—C7	−1.1 (3)	C11—C12—C13—C14	−2.3 (3)
C4—C5—C6—C7	−178.5 (2)	C17—O4—C14—C13	−4.7 (3)
O1—C6—C7—C8	−5.8 (4)	C17—O4—C14—C9	174.2 (2)
C5—C6—C7—C8	173.2 (2)	C12—C13—C14—O4	179.2 (2)
C6—C7—C8—C9	179.0 (2)	C12—C13—C14—C9	0.4 (3)
C7—C8—C9—C14	14.7 (4)	C10—C9—C14—O4	−177.1 (2)
C7—C8—C9—C10	−165.6 (2)	C8—C9—C14—O4	2.6 (3)
C15—O2—C10—C11	−0.4 (3)	C10—C9—C14—C13	1.8 (3)
C15—O2—C10—C9	178.7 (2)	C8—C9—C14—C13	−178.6 (2)
C14—C9—C10—O2	178.7 (2)		

### Hydrogen-bond geometry (Å, °)

**Table:**

*D*—H···*A*	*D*—H	H···*A*	*D*···*A*	*D*—H···*A*
C3—H3A···O2^i^	1.00 (2)	2.45 (3)	3.369 (2)	157.8 (17)
C7—H11B···O4	0.97 (3)	2.31 (2)	2.835 (2)	113.1 (18)
C17—H17B···O4^ii^	0.99 (2)	2.46 (2)	3.337 (3)	148 (2)

Symmetry codes: (i) *x*+9/4, −*y*+9/4, *z*+1/4;  (ii) −*x*+1/2, −*y*, *z*−1/2.
